# Parents’ perceptions of their children’s physical activity during the COVID-19 pandemic

**DOI:** 10.1186/s12889-022-13829-y

**Published:** 2022-08-01

**Authors:** Emma Ostermeier, Patricia Tucker, Danielle Tobin, Andrew Clark, Jason Gilliland

**Affiliations:** 1 Health and Rehabilitation Sciences, Faculty of Health Sciences, The University of Western Ontario, London, ON Canada; 2 Child Health and Physical Activity Laboratory, School of Occupational Therapy, The University of Western Ontario, London, ON Canada; 3 Human Environments Analysis Laboratory, Department of Geography and Environment, The University of Western Ontario, Social Science Centre, Rm 2333-1151 Richmond Street Western University, ON N6A 3K7 London, Canada; 4 School of Occupational Therapy, Faculty of Health Sciences, The University of Western Ontario, London, ON Canada; 5 Children’s Health Research Institute, N6C 2V5 London, ON Canada; 6 Department of Epidemiology & Biostatistics, The University of Western Ontario, London, ON Canada; 7 Department of Paediatrics, The University of Western Ontario, London, ON Canada; 8 Department of Geography and Environment, The University of Western Ontario, London, ON Canada; 9 School of Health Studies, The University of Western Ontario, London, ON Canada; 10 Lawson Health Research Institute, London, ON Canada

**Keywords:** Physical activity, Children, Interviews, Parent, Coronavirus, Pandemic, Qualitative

## Abstract

**Background:**

COVID-19 has drastically changed the everyday lives of children, including limiting interactions with peers, loss of regularly organized activities, and closure of schools and recreational facilities. While COVID-19 protocols are in place to reduce viral transmission, they have affected children’s access to physical activity opportunities. The purpose of this study was to understand how COVID-19 has affected children’s engagement in physical activity and to identify strategies that can support children’s return to physical activity programming in public places.

**Methods:**

Parents of past participants in the Grade 5 ACT-i-Pass Program in London, Ontario, Canada were invited to participate in a semi-structured interview online (in November and December 2020) via Microsoft Teams. The script was comprised of questions about their child’s physical activity levels (before, current, and anticipated following COVID-19), lifestyle changes due to COVID-19, and what service providers can do to assist children’s return to public programming. Interviews were transcribed in Microsoft Teams, reviewed by a member of the research team, and analyzed in NVivo 12 using thematic analysis.

**Results:**

Twenty-seven parents participated in an interview. Four themes and two subthemes were identified during analysis: (1) modifications to everyday life (a. activity options available and b. altered social environment), (2) safety in public spaces, (3) accessibility of activities, and (4) utilizing outdoor spaces.

**Conclusions:**

COVID-19 protocols have decreased children’s physical activity levels due to the loss of their regular activities, recreational spaces, and peer support. Implementing facility and activity-specific health protocols, providing outdoor activity options, and offering a variety of activity types, times, and locations are three strategies recommended by parents to help facilitate their children’s return to public recreational places. Due to the negative consequences of physical inactivity on children’s health and well-being, service providers need to implement programming and safety protocols that support children’s engagement in physical activity throughout the remainder of, and the years following, the COVID-19 pandemic.

**Supplementary Information:**

The online version contains supplementary material available at 10.1186/s12889-022-13829-y.

## Introduction

Physical activity benefits children’s physical and psychosocial health by positively impacting their cardiometabolic health, bone strength, adiposity, depressive symptoms, anxiety, and cognitive functioning [1–3]. Despite consistent research demonstrating the advantages of physical activity on children’s health outcomes, physical inactivity remains a public health concern, with 61% of Canadian children (aged 5 to 17 years) not achieving the 60 min of daily moderate-to-vigorous intensity physical activity recommended in the *Canadian 24-Hour Movement Guidelines for Children and Youth* [4].

The COVID-19 pandemic has created a new set of challenges for families due to the public health measures countries have implemented to deter transmission of the virus, [5] including physical distancing, discouraging social gatherings with people outside of the immediate household, and stay-at-home orders [6, 7]. While the COVID-19 protocols protect families from viral transmission, the lifestyle adjustments associated with the COVID-19 protocols have negatively impacted children’s physical activity and sedentary behaviours [8–10]. For instance, peers have been identified as an important influencer of children’s physical activity by providing support and encouraging engagement in activities; [11] however, social gatherings with individuals outside of their household have been discouraged and, in some places, schools have been closed. Furthermore, children’s environments have changed with many indoor recreational facilities, such as gyms, arenas, and community centres, either being closed or providing a limited number of spaces and programming options. In Canada, indoor recreational facilities are particularly important in the winter, when inclement weather can hinder children’s use of outdoor physical activity spaces. Without recreational facilities, children have minimal access to organized activities, ultimately resulting in lower physical activity participation [12].

As COVID-19 is an unprecedented time, it is unclear how national, provincial/state, and local COVID-19 regulations have affected children’s physical activity, and, if and how parents intend to return their children to public recreational spaces as the pandemic subsides [9]. Due to children’s decline in physical activity and increase in sedentary activities, there is fear that these changes in behaviours will have lasting effects on children’s health behaviours; [13] therefore, it is important to find ways to facilitate children’s return to recreational programming.

The purpose of this study was to explore parents’ perceptions of their children’s physical activity participation during and following the COVID-19 pandemic. Specifically, this study sought to: (1) understand how COVID-19 has affected children’s engagement in physical activity; and (2) identify strategies that can support children’s return to physical activity programming in public places (e.g., community centres, pools, arenas, and small businesses) during and following the COVID-19 pandemic.

## Methods

### Study design

This study recruited participants from a larger, longitudinal evaluation of the Grade 5 ACT-i-Pass, a physical activity program in London, Ontario, Canada that offers children in grade five free organized and drop-in activities at participating recreational facilities (e.g., YMCA, Boys and Girls Club, recreation centres) [14]. More specifically, each grade five student is provided with a pass which grants them, and a friend or sibling, access to a variety of recreation facilities in London. This program is a collaborative undertaking by the municipality, researchers and community organizations that aims to reduce financial barriers to physical activity opportunities and increase families’ knowledge of the physical activity opportunities within London [14]. It is key to develop interventions that target school-aged children (8 to 12 years) as effective health promotion programs can improve children’s quality of life [15], can prevent the declines in physical activity children tend to experience when they transition into adolescence [16], and can cultivate healthy habits that can translate into adulthood [17]. This qualitative study consisted of semi-structured interviews with parents regarding their children’s physical activity during the COVID-19 pandemic and recommendations for returning to recreational programming following the pandemic. This study protocol was approved by Western University’s Non-Medical Research Ethics Board (REB #103954).

### Participants and recruitment

 Eligible participants for the study included parents that had a child who was enrolled in a grade 5 through 8 class (ages 9–14 years) during the 2020–2021 school year and was currently or previously registered in the ACT-i-Pass program. Two forms of recruitment were used: (1) parents of grade 5 children were invited to participate in the study after registering for the ACT-i-Pass; and (2) parents of children in grades 6 through 8 were invited to participate through the ACT-i-Pass Newsletter, which was publicly available online and emailed to previous registrants. Parents were provided with a letter of information describing the project and asking if they consented to be contacted by the research team to participate in the study. Of the parents who consented, purposeful sampling was used to recruit a diverse group of parents that could provide a variety of experiences and perspectives on the COVID-19 pandemic [18]. Survey data collected during study recruitment was used to consider a variety of parent and child characteristics during sampling, including gender (boy/man, girl/woman, self-identify), child’s grade (grades 5–8), ethnicity (White, Black, East or Southeast Asian, Indigenous, Latin, Central or South American, Mixed), immigration status (born in Canada, ≥ 5 years, < 5 years), income (low, middle, and upper/upper-middle; income group were divided into tertiles based on the median family income of all the dissemination areas in London, Ontario [*n* = 570]), the number of days per week their child accumulate at least 60 min of physical activity (0–2 days, 3–5 days, 6–7 days), school mode during COVID-19 (in-person learning, full remote learning, homeschooling), recreational facility use during COVID-19 (indoor and outdoor, only outdoor, no public facilities), and interactions with extended family and friends during COVID-19 (able to interact with anyone, able to interact with anyone with physical distancing, only interacting with immediate family and/or close friends).

### Context

Interviews were conducted in November and December 2020 (i.e., during the COVID-19 pandemic) with parents in the mid-sized Canadian city of London, Ontario. Located in the Southwestern region of the province, London residents experience seasonal changes in the weather that can impact the types of activities children can partake in during different times of the year [19]. The interviews were collected during late fall into the winter, when temperatures ranged from − 6 to 7 degrees Celsius and had 92.4 and 343.4 millimetres of snow in November and December, respectively. London contains around 600 outdoor amenities, such as outdoor aquatic facilities, sports fields, playgrounds, and gardens, as well as 24 community recreational centres, including pools, arenas, and gymnasiums [20].

At the time of these interviews, London, like other regions of Canada, had already endured the ‘first wave’ of the COVID-19 virus and was experiencing a ‘second wave’ with record-breaking increases in COVID-19 cases. Children were able to access many indoor and outdoor recreational places, but with strict protocols including mask and physical distancing mandates, limitations to the number of programming times, and restricted occupancy [7]. Organized sports and activities were either unavailable or coaches and program coordinators had to adapt activities to meet these protocols (e.g., physically distanced practices without games). Designated physical activity times in schools (i.e., recess and physical education) were offered, but the curriculum was adapted to limit potential COVID-19 spread, such as moving physical education classes outdoors and limiting recess times to grade cohorts.

### Data collection: interviews

The interviews were semi-structured and lasted 30 to 45 min. Conversations were guided by an interview guide developed by members of the research team that contained six main questions and prompts (Additional file 1). The guide consisted of questions about physical activity levels (before, current, and following COVID-19), lifestyle changes due to COVID-19, and what service providers can do to assist children’s return to public programming. To meet COVID-19 safety protocols, interviews were conducted via Microsoft Teams. Prior to the questions, participants were asked if they consented to be recorded. They were also informed that there were no wrong answers and that they are welcome to skip questions they were not comfortable answering. Two members of the research team (i.e., moderators) conducted the interviews. Both moderators have previous experience conducting qualitative research, including facilitating interviews, developing codes, and synthesizing data into overarching themes. Following the first week of interviews, moderators watched the recording of an interview conducted by the other moderator to improve the consistency of the interviews. Interviews were undertaken until saturation was reached [21].

### Data analysis

Transcripts were created by the program Microsoft Streams following the completion of the interview and were reviewed for accuracy by a member of the research team. Participants were anonymized by converting their names to a unique identifier, which includes the participant number, grade of child, and gender of child. Reviewed transcripts were imported into QSR NVivo 12 and an inductive analysis directed by Braun and Clarke’s [22] guide for a reflexive thematic analysis was conducted by the two moderators. A reflexive approach to the analysis was used to recognize the influence of the researchers’ positionality and knowledge of the subject matter on the themes produced from the data [23, 24]. First, the moderators familiarized themselves with the data by reviewing the transcripts and noting preliminary thoughts on the data and ideas for coding. Subsequently, transcripts were coded in entirety to identify data of interest; codes were grouped into potential themes that were reviewed to determine if the themes should be combined, separated, or removed. Once the themes were finalized, the themes were defined and named, and the report was produced.

To add rigour to the findings, Guba and Lincoln’s [25] criteria for trustworthiness (i.e., credibility, dependability, transferability, and conformability) were used to assess the quality and accuracy of the themes created from the data. Reflexive activities, such as recording thoughts and interpretations of the data via reflexive journaling and discussions amongst the researchers, were conducted [26]. Additionally, critical friends were also used to encourage the researchers to reflect upon their interpretation of the data and to consider alternative explanations from other members of the research team [27]. Critical friends were beneficial as this process considers that the themes derived from the data may differ between researchers in order to develop plausible findings based on the data [27].

## Results

Of the 5,674 children who were enrolled in the ACT-i-Pass between 2017 and 2020, 92 of their parents agreed to take part in an interview. Twenty-seven parents were interviewed to reach saturation. Most of the respondents were women (*n* = 23) and there was a relatively equal distribution of low (*n* = 9), middle (*n* = 6) and upper (*n* = 10) income families. A full description of the parent and child characteristics can be found in Table 1.

**Table 1:** Parent and child characteristics from the sample population (*n* = 27)

Characteristic	n (%)
Parent Gender	
Man	4 (14.8)
Woman	23 (85.2)
Median Family Income (MFI)	
Low (< $70 000 CAD)	9 (33.3)
Middle ($70 000 – $94 999 CAD)	6 (22.2)
Upper/upper-middle (> $95 000 CAD)	10 (37.0)
Immigration Status	
Born in Canada	20 (74.1)
≥ 5 years	3 (11.1)
< 5 years	3 (11.1)
Child Grade	
5	10 (37.0)
6	7 (25.9)
7	7 (25.9)
8	2 (7.4)
Child Gender	
Boy	13 (48.2)
Girl	12 (44.4)
Child Ethnicity	
White	13 (48.2)
Black	2 (7.4)
East or Southeast Asian	3 (11.1)
Indigenous	1 (3.7)
Latin, Central or South American	1 (3.7)
Mixed	4 (14.8)
Child Physical Activity^a^	
Low (0–2 days)	6 (22.2)
Moderate (3–5 days)	10 (37.0)
High (6–7 days)	9 (33.3)
Child Schooling Model	
In-person learning	18 (66.7)
Full remote learning	6 (22.2)
Homeschooling	1 (3.7)
Data was collected from a survey disseminated to families with the interview consent form. ^a^ parents were asked, “over the past 7 days, on how many days was your child physically active for a total of at least 60 minutes per day?”. Some responses do not add up to 27 due to missing data	

The analysis of the interviews resulted in four themes and two subthemes that describe the influence of the COVID-19 pandemic on children’s physical activity: (1) modifications to everyday life (a. activity options available; and, b. altered social environment), (2) safety in public spaces, (3) accessibility of activities, and (4) utilizing outdoor spaces.

### Modifications to everyday life

Parents attributed changes in their children’s physical activity levels to the COVID-19 protocols modifying the places, activities and people children can play with on a daily basis. This theme was illustrated by two subthemes: (a) activity options available; and, (b) altered social environment.

#### Activity options available

Parents described their children as having difficulties engaging in physical activity during the COVID-19 pandemic due to the lack of activity options available. To meet the COVID-19 protocols, many recreational facilities alternated between remaining open at a lower capacity or closed, which “affected their level of activity indoors and being able to be in community center type settings” (P24_Gr6_Unknown). As a result, many of the children’s regular activities were “cancelled due to the restrictions” which “clearly had an impact [on his physical activity] not having this scheduled time to go.” Parents also recounted that children were not getting their regular physical activity during school recreational times, as “it’s not physical education like you or I would know it” (P4_Gr8_Boy). As a result, children “missed out on those kinds of, you know, extra fun activities that they normally would have done” during the pandemic (P3_Gr6_Girl).

With many indoor recreational facilities closed and organized activities cancelled, children became dependent on activity options at home. The additional time at home placed pressure on parents to engage their children in physical activity. As one parent described, “It’s really kind of on us as parents to kind of make sure we get our kids active and enjoy it, right? Particularly because my kids are doing online schooling, it’s so much time in front of the computer that we’re trying to offset that for sure.” (P22_Gr7_Girl). One strategy that parents used to create physical activity opportunities at home was to purchase exercise and sports equipment, such as basketball nets, indoor exercise bikes, and trampolines, to encourage children to engage in physical activities and try new activities. Parents also organized activity breaks during school hours to compensate for the loss of recess and physical education classes, including “jumping jacks and race up and down the hallway, do push-ups and sit-ups, and stuff like that” (P14_Gr8_Boy).

To alleviate the loss of organized activities, select recreational programs developed virtual programming for children. Some parents felt the virtual programming was a valuable resource for maintaining their children’s activity levels at home, particularly among children who struggle with the COVID-19 restrictions. However, some children did not respond well to the online activities, as they “didn’t enjoy it as much” (P5) and “you don’t have spring-loaded floors or equipment in a house, so the zoom did not work in that situation” (P4_Gr8_Boy).

Parents also recounted that their children’s activity preferences changed during the pandemic. Walking and biking became popular forms of physical activity for families. Unstructured play also became more common during the pandemic, with one parent explaining, “I would say over the summer she was very, very active; it wasn’t as much organized sports at that time. It was more walking and playing with sisters and bike riding and things like that” (P25_Gr7_Girl). This also included imaginative play where children created role-playing games:

“They were really imaginative games where somebody was the guard and somebody else was collecting pinecones and other things for the food at the base, and somebody else was recruiting other guards and someone was a bad guy that type of game.” (P2_Gr7_Girl)

While the public health protocols were enforced, parents suggested that recreational facilities provide information on the programming available, the COVID-19 protocols in place, and the process of enrolling in programming. Parents described being unaware of the opportunities available at recreational centers and recommended that businesses and community centers improve the promotion of their programs, as “more people would take advantage of it [available programs] if they knew” (P19_Gr6_Boy). There were also recommendations to encourage unstructured play following the pandemic, including “real bike lanes” (P13_Gr5_Boy) and drop-in activity options “like a dodgeball thing for them” (P15_ Gr7_Girl).

#### Altered social environment

Throughout the pandemic, the ability to engage in activities with friends and family affected children’s physical activity levels. Parents of children who were able to play with friends felt the pandemic “hasn’t [affected her physical activity] because the people in a bubble are the ones that she would have played with anyway and so she’s not missing much on that part of it.” (P23_Gr7_Girl). Alternatively, the parents that enforced greater restrictions described difficulties engaging their children in activities due to the COVID-19 protocols, as one parent explains, “He really wants to go out with his friends, but because most families don’t allow the children to go out…I allow him to go out to play basketball, but nobody plays with him. Then he just plays once or two times, then he comes back home” (P17_Gr7_Boy). The loss of peer interactions was described as difficult for their children and was attributed to their limited physical activity participation as they were “not seeing her friends and doing all the things they used to do” (P8_Gr5_Boy). With fewer opportunities to be active with peers, children engaged in more activities with parents and siblings, such as, “a few family bike rides that we probably wouldn’t have done honestly if my husband and I were both working regular hours” (P27_Gr7_Girl).

As the COVID-19 cases declined in the summer, parents mentioned they extended their bubble to include other families. This included families either partnered with “one family we interact with, and he still plays with every day and there’s three boys in that family” (P20_Gr5_Girl) or their child had “a few close friends that we have over from time to time” (P2_Gr6_Boy). One parent recounted, “it was hard to recognize how much he missed them [friends] until I saw him playing at the park with other kids and running and it was really a stark reminder of how necessary those social exchanges are at his age.” (P2_Gr7_Girl). As a result, parents requested that service providers develop activities that “get him to be able to socialize and be active” (P11_Gr6_Boy) as children “need to talk to someone their age.” (P26_Gr5_Boy).

### Safety in public spaces

Parents’ perception of the adequacy of the COVID-19 health measures and their experiences at recreational facilities influenced the places where their children were allowed to be active. Some parents were comfortable in returning their children to programming once COVID-19 restrictions lessened, explaining that their decision to return their children to activities was due to programming being “a pretty calculated risk with all of the safety measures in place…I would rather her be active but be active in a safe, controlled environment and have that possibility for exposure than not be active at all” (P10_Gr5_Girl). Parents also described returning their child to programming as physical activity improved their child’s well-being, including “attitude was better with sports” (P22_Gr7_Girl) and “sports helps with focusing on schoolwork” (P14_Gr8_Boy).

Some parents commented that the current protocols (e.g., mask-wearing mandates, hand sanitizer accessibility, tracking systems, staggering the flow of people in and out of buildings) in place at facilities were sufficient for their children to safely return. Return to activities was also facilitated by program coordinators making the appropriate adaptations to activities to increase the safety of programming. For instance, parents noted that community centers modified swim lessons to meet the COVID-19 protocols by “limited the number of people” and “a parent has to come into the pool with the child so that they’re the ones touching the child and doing the hands-on if anything needed to be corrected in the lesson” (P3_Gr6_Girl), and that made them comfortable to register their children.

 Conversely to the parents who described the current protocols as sufficient, many parents expressed concern about the safety of public spaces and returning their children to recreational programming. Some parents commented that the health protocols in place do not give them comfort as they are not realistic for their child’s activities:

“He [public health official] was speaking and he was talking about, you know, keeping a two-meter distance playing hockey, right? I don’t know if the guy’s never been to a rink in his life, but that’s not going to happen, right? Like you put a bunch of 12-year-olds or any age really. You put them on a rink and drop a puck, they’re going to bump into each other, right?” (P6_Gr5_Boy)

Additionally, COVID-19 protocols were characterized as challenging for children to follow or limiting access to programming, particularly for those with differing abilities:

“Well, I would say I have avoided activities for [child’s name] where he would have to physically distance or wear a mask because it would be really difficult for him to adhere to those policies. He has a really hard time maintaining physical distance from people and he has some mental health issues that make the mask-wearing challenging. So, I don’t think it wouldn’t be very successful for him if he had to [wear a mask].” (P2_Gr7_Girl)

In some cases, there are no additional protocols that would make parents feel safe. Some parents explained that they were concerned about all public spaces outside of their home and minimized their time in public spaces. Alternatively, some parents opted to wait until their family is vaccinated: “I’m thinking like nothing was really guarantee or safer unless you know everybody or at least we have the vaccine, right? So, I’m thinking once I have the vaccine, I should be able to act more normal” (P9_Gr6_Girl).

Parents were also concerned about the safety of the recreational facilities due to the information available to the public about the transmission of the COVID-19 virus. Parents described their decision to not return their children to recreational facilities as influenced by “a lot of cases out in the community” (P6_Gr5_Boy), and the need for “some concrete thing they [service providers] did to lower the risk” (P9_Gr6_Girl). The little information about the COVID-19 cases amongst children in organized sports programming was also highlighted as a concern:

“I haven’t heard anything bad in the media so I feel like if things tend to be going well, but I would just feel more comfortable knowing that the soccer team that’s been playing together for eight months has never had a case and that things are going well in that team and that would make me feel a lot more comfortable.” (P25_Gr7_Girl)

As a result, one parent suggested that the media have “news stories about here’s what this team is doing or here’s what that’s team’s doing and knowing that it’s safe, knowing that it’s safe to be out there and they’re enjoying themselves and they’re distancing” (P25_Gr7_Girl). There were also concerns about the other people attending programming not following the protocols set by the service provider: “But I looked across and there was a group of guys like you know our age and they’re all standing shoulder to shoulder and chatting… if there is spread then the kids will lose their sports, right?” (P6_Gr5_Boy). Although children are interested in “something structured or they want to go somewhere and do something” (P13_Gr5_Boy), some parents have decided to not allow their children to partake in select activities as it is not possible to know where people have been and whom they have interacted with. As explained by Parent 25 [Gr7_Girl], “I think some of the teams were meeting in person, maybe they shouldn’t have been, but in any case, it made us really, really nervous. We’re trying to do whatever activity we could at home with our daughter.”

To increase the safety of public spaces, health measures have been put in place by the provincial and federal governments; however, parents believed that service providers will need to make “a lot of changes to how they’ll be able to structure those activities if COVID still going on over the winter when a lot of outdoor sports have to move indoors.” (P25_Gr7_Girl). As they adapt activities, service providers will need to find ways to support children’s return to activities in public spaces. It was suggested that facilities “need a very big space where people can actually be six feet apart” (P9_Gr6_Girl), “making sure the children continuously wash their hands or sanitize their hands” (P14_Gr8_Boy) and creating a “reserve a spot kind of thing” to limit the number of children at an activity (P7_Gr6_Boy). Parents also recommended that service providers provide “four or five classes on a Saturday, then it would still give everybody the option to be able to do it without going over the numbers of people that are allowed in the class” (P24_Gr6_Unknown) and “need to have more clear guidelines” (P9_Gr6_Girl); specifically, creating consistent protocols across facilities to help with children’s transition to the new rules at recreational facilities.

### Accessibility of activities

Parents reported that financial circumstances and neighbourhood features affected their children’s access to physical activity opportunities during the pandemic. For instance, a parent felt her child’s physical activity levels reduced since she was unable to register them for activities, stating “unless I can find something for him that I can afford… The opportunity is not there, is just not there or it’s just too expensive” (P14_Gr8_Boy). When asked to expand, Parent 14 [Gr8_Boy] explained that while registering for swimming lessons, “they want private lessons, which is like more than double the cost, so instead of $60 they want $160 for 8 weeks, which is quite pricey when you have two children.” Parents also commented on activities not having the same value as “you only got half the time for the same price, ‘cause typically they were open for 3 hours and you paid $10 where you pay $10 now for an hour and a half” (P11_Gr6_Boy). As a result of financial limitations, some parents felt that their children’s activity options were limited: “financially we wouldn’t have the money right now to invest in like teaching him new activities like skiing or other things, but it just feels right now like just maintaining his current activity level over the winter would be, you know” (P2_Gr7_Girl).

The locations of activities were also highlighted as important for children’s physical activity during the pandemic. Parents frequently described the benefits of nearby outdoor recreational facilities on physical activity participation. For instance, a parent explained, “I live about a 20-minute walk from the new trail, so we do have the access to that in the summer and we’ve certainly used a lot of it” (P4_Gr8_Boy), and “we’re across the street from a forest and to the left is a field with trails through both of them, so they like to take our neighbour’s dog and walk around through the trails” (P14_Gr8_Boy). Conversely, families that lacked recreational places near their homes described challenges in maintaining their children’s physical activity throughout the pandemic. This was particularly a struggle for families that lived in high-density communities (e.g., apartments or homes that lacked backyards): “because we live in an apartment, so the only things we can do actually, just go to the swimming pool at the registration [time], go just for a little walk around our buildings… Yeah, this is the only option actually” (P26_Gr5_Boy).

Moving forward, parents want additional local activity options to reintroduce their children to public physical activity programming as, “I think location is so important for families, especially those you know without vehicles or multi vehicles” and suggested that local programming should be allocated to “more low-income neighbourhoods, you know, if their kids are able to walk to the facilities or bike or a quick bus ride” (P27_Gr7_Girl). For families that do not have programming and limited spaces for activities at home, parents are looking for activities that can be adapted to the recreational spaces available at or near their homes. For instance, parents wanted activities that “consider our restrictions as well, because we live in an apartment and this apartment is only two bedrooms or you can say like 200 or up to 300 square feet” (P26_Gr5_Boy) and requested activities that could be modified for small spaces. Parents also believed that free physical activity opportunities could support children’s physical activity participation following the COVID-19 pandemic, as many families have gone through financial hardship due to business closures and downsizing.

### Utilizing outdoor spaces

Parents described the outdoors as an important setting for children to engage in physical activity during the pandemic. As many organized sports were not a viable option due to the public health protocols, families tried to take advantage of outdoor activities. Some parents identified the pandemic had “reconnected us, I think, to being outside a lot more ‘cause that’s the things that we could do” (P3_Gr6_Girl), due to indoor recreational programming has been unavailable throughout the pandemic. As children cannot partake in their normal afterschool activities, outdoor spaces were seen as an important outlet for children’s physical activity: “[He] is always in sporting activities, like organized team sport activity, during the summer, so that was cancelled, so that clearly had an impact [on his physical activity] …So, we did spend a lot of time outside” (P7_Gr6_Boy).

Even when programming in indoor facilities returned, some parents did not enrol their children in activities as they felt that outdoor spaces were safer: “I think also having more opportunity to be outdoors versus indoors ‘cause it feels much safer having groups of children outdoors to me than in an enclosed space.” (P14_Gr7_Girl). As they felt more comfortable with outdoor spaces, parents allowed their children to socialize with other children. Specifically, parents described the outdoors as spaces that allowed children to follow the public health protocols. For instance, one parent mentioned, “this summer things open up, but we didn’t do a lot of indoor or structured things, but they did get out with their friends a lot in the summer because they could bike and do things outside that was still physically distance” (P18_Gr5_Girl).

Outdoor spaces were ideal during the summer as they “increased it [children’s physical activity] immensely, so the weather being nice that being able to go outdoors, right?” (P3_Gr6_Girl). However, as summer transitions to winter, parents foresaw their children’s physical activity “will have a negative impact, especially during the winter because it’s going to be a little bit harder to go outside” (P10_Gr5_Girl). Predominantly, the parents of children who were not born in Canada recounted their children having difficulties with outdoor activities during the winter months: “he doesn’t like playing with the snow too much, and for him, it is a little bit difficult, because, uh, we just got to Canada for few years. He is not growing up in Canada. He is not adapted to the cold environment” (P17_Gr7_Boy). While winter weather was described as a deterrent by many parents, some parents noted that snow facilitated activities: “[my child] actually, got to play with some random neighbours that she hasn’t seen for a long time, so snow actually brings some activities back like snowball fight like building, you know whatever ice mountain horse snowman, right?” (P9_Gr6_Girl).

Outdoor recreational spaces are viewed as desirable spaces for physical activity as the public health measures subside. Parents suggested service providers utilize the outdoor recreational facilities in the city for their programming, including “having some outdoor physical classes set up, like, maybe a public kind of skating, you know, outdoor skating class or skiing” (P1_Gr5_Girl). Outdoor physical activity opportunities were believed to be the advantageous and safest approach for returning children to public spaces with peers.

## Discussion

This study explores parents’ perceptions of their children’s engagement in physical activity during the COVID-19 pandemic and identifies strategies to support children’s return to physical activity programming in public places. While public health measures offered protection from COVID-19 transmission, parents indicated that the protocols negatively impacted their children’s health behaviours. Consistent with local [28], national [9, 29] and international findings [13, 30–32], many parents reported their children’s physical activity declined during the COVID-19 pandemic; the change in activity levels was primarily credited to the loss of organized activities and limited physical activity opportunities. To meet the government’s health and safety measures, recreational facilities were either closed or were running at low capacity, disrupting children’s regular after-school programming [33]. Participation in organized activities, such as sports, recreational programming, and physical education classes, has been associated with children achieving higher physical activity levels and greater chances of children meeting the physical activity guidelines [34]. Organized activities can increase children’s chances of meeting the physical activity recommendations by comprising of structured drills and games that may be a higher intensity than unstructured activities. Team sports and recreational programming also have a coach or facilitator that may support greater participation in activities through inclusion and encouragement [35], and children can receive social support from peers during activities [36]. Without physical activity opportunities, children resorted to screen-based activities for entertainment [29]. The greater exposure to screen-based devices and lack of social connectedness during the pandemic negatively affected the mental [37] and emotional health of children [38]. As COVID-19 regulations lessen, the return of organized activities is an important strategy for parents to engage their children in physical activity. Social interactions with peers can lead to improved psychosocial health and emotional well-being, including higher self-esteem, improved social skills, fewer indicators of depression and greater confidence [39]. To facilitate children’s return to public recreational places, a variety of programming options are needed. The availability of sports and recreational programming (e.g., types of activities and multiple time slots for activities) contributes to greater participation in organized physical activity [40]. Offering various activity times to account for the limited spaces available at a single activity and providing a variety of activity options to meet children’s activity preferences can support children’s return to physical activity programming.

In addition to the activity options available, parents commented on the difficulties accessing activities available during the pandemic. Primarily, parents felt that the programming options available during the pandemic at facilities were not sufficiently publicized. To create accessible programming, distributing promotional materials that inform and explain the available activity options to the target audience is critical [41]. Parents also felt that the financial constraints decreased the feasibility of physical activity programming during the pandemic, as some activities increased in price due to demand and limited activity availability. COVID-19 has been a time of financial hardship for many families with high rates of job instability due to layoffs and downsizing of businesses. Beyond membership costs and league fees, there are additional fees associated with physical activity participation, such as equipment and transportation, which can act as a significant barrier for children [42]. When families are struggling to pay for their necessary expenses, they are unable to afford physical activity programs [43]. To assist children’s return to recreational programming, service providers need to develop additional promotional materials to inform families about the physical activity opportunities in their community and how children can safely engage in physical activity [44]. Also, providing low-cost physical activity opportunities for children, such as free drop-in programs and discounted membership rates for lower-income households, can help support families who experienced new or exacerbated financial hardships during the pandemic.

Although there were concerns about the potential spread of the virus at recreational facilities, some parents emphasized the importance of physical activity on the physical, mental and social well-being of their children when justifying the decision to return their children to physical activity programming. While there was excitement about returning to their regular activities, some parents were apprehensive about the safety of recreational places, particularly indoor recreational facilities, and want to ensure facilities were taking the necessary precautions before their children returned to activities. For indoor recreational places, the enforcement of practical protocols by service provider staff, such as COVID-19 screening at facility entry, limiting capacity, additional cleaning and sanitization of the facility and equipment, and customized rules for specific activities in addition to the general facility rules, can help mitigate the risk of COVID-19 spread [45, 46]. Additionally, measures can be taken to make outdoor spaces safer by allocating additional road space to cyclists and keeping parks and green spaces open to help provide socially-distanced spaces for children to be active [9].

Alternatively, some parents felt that their child maintained their physical activity levels during the pandemic by engaging in new or alternative activities. Parents credited the availability of outdoor spaces facilitating their children’s physical activity. Children who have parks and recreational facilities within walking distance of their home tend to be more active,[47] and utilizing parks and outdoor spaces have also been associated with children meeting the daily physical activity recommendations [48]. Mitra et al. (2020) proposed that outdoor physical activity has become easier for children during the COVID-19 pandemic due to stay-at-home orders decreasing traffic on neighbourhood streets and allowing for parental supervision [49]. Outdoor physical activity facilities, such as parks and trails, have become important during the COVID-19 pandemic as it provides a safer space for physical activity with lower transmission risk. These spaces have become particularly beneficial for children who reside in high-density housing as their homes lack adequate space for activities [49]. In future waves of the pandemic, it is critical outdoor spaces remain open to ensure children have an opportunity to be active. Service providers should also increase the number of outdoor programs available to support children’s physical activity.

Virtual activities organized by their coaches or program coordinators were also beneficial for some children throughout the pandemic. While virtual activities can not necessarily replace children’s regular programming due to equipment restrictions in homes and the lack of social support from peers, it provides at-home programming to families who are hesitant to use recreational facilities. Understanding how to adapt activities to available spaces is important for children’s physical activity participation [48], and can promote resilience which is critical as we progress through the pandemic [48]. Virtual activities can be particularly beneficial for children who live in rural communities with limited access to recreational opportunities. Online physical activity programming can also offer activity options for children with disabilities who may struggle with social distancing and mask mandates at facilities. Children with special needs had a worsened quality of life during the pandemic, including greater anxiety, poorer emotional well-being, additional screen time, and overuse of technological devices [50]. Telehealth is considered an appropriate method for engaging children with special needs in healthy behaviours in a safe, socially-distanced manner [51]. With the loss of school and child-care, additional pressure was placed on parents to support their children’s health [38]. In response, service providers should support families by continuing to offer virtual activity options to families as the pandemic subsides since it can act as an introductory step towards returning to recreational programming. Activities should ideally be flexible so they can be tailored to a child’s unique needs and the equipment available in each household. To increase the accessibility of at-home activity options to families who may have limited access to the internet, service providers can also provide a printable list of activities that can be shared with parents to improve their capacity to create effective and engaging activity times in their homes.

**Fig. 1:**
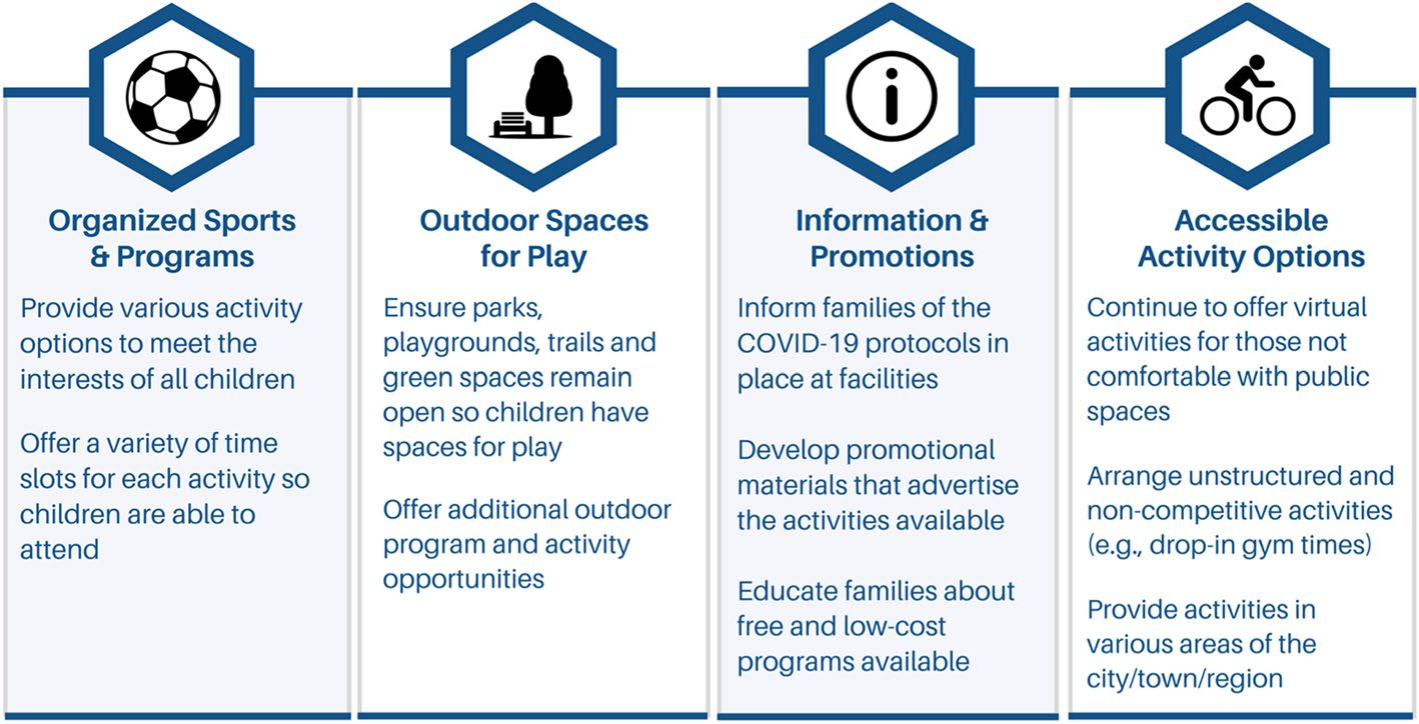
Parent-informed recommendations and strategies for returning children to public recreational facilities and programs

To maintain physical activity levels, families had to adjust their regular activities based on the opportunities that were available to them [33]. As the COVID-19 pandemic subsides, the long-term effects of children’s activity choices during the pandemic will need to be taken into consideration during the planning of recreational programming. Teare and Taks (2021) propose that previous global events have indicated that children’s physical activity preferences will change due to the lifestyle changes they experienced during COVID-19 [52]. In the current study, parents mentioned that their children had a change in their activity preferences throughout the pandemic, with many parents specifying that their children engaged in more unstructured play. As children have spent a large amount of time in unstructured or self-organized activities versus their usual organized activities, some children may prefer unstructured forms of physical activity following the pandemic. This may be due to children having negative peer interactions or experiences in competitive environments, enjoying the unstructured and less competitive activities, or preferring involvement in various activities as opposed to specializing in one activity [53, 54]. In addition to the health benefits associated with physical activity, unstructured, play-based activities offer children an opportunity to be creative and collaborate with peers, benefiting their cognitive and social development [55]. Unstructured play is particularly beneficial for children with disabilities by proving them with flexible activities that offer an opportunity to make connections with their peers, improving their social competence [56]. Providing children with unstructured physical activity opportunities (e.g., drop-in gym, swim, and skate times) and developing more non-competitive activity options can potentially be a beneficial way to support children’s return to public spaces. An overview of the parents’ recommendations and strategies to support children’s return to public recreational programs is provided in Fig. 1.

### Limitations

While these findings contribute valuable insight into parents’ perspectives of their children’s physical activity during and following the COVID-19 pandemic, there are limitations to this study. This study took place in London, Ontario, Canada. Ontario experienced frequent stay-at-home orders, including recreation centres being closed or at limited capacity and mask-wearing mandates being consistently enforced. As the COVID-19 protocols may be stricter or less stringent than the COVID-19 protocols in other regions and counties, the generalizability of the findings is limited. Additionally, most participants were selected from a list of parents who consented to be contacted about the study, there is the potential that volunteer bias may have been introduced to the study as the parents who agreed to participate may have a particular interest or may be advocates for physical activity. The participants were also predominantly women and mothers; consequently, the data collected may lack the viewpoint of other parental figures (e.g., father, foster parent, grandparent, other parent in the household).

## Conclusions

The findings from this study provide insight into how the COVID-19 pandemic has influenced children’s physical activity participation and offer suggestions for how service providers can support children’s return to physical activity programming in public spaces. Examinations of children’s health behaviours during the pandemic found that children’s physical activity has declined [9]; accordingly, the findings from this study can be employed by service providers and community stakeholders to promote and encourage enrollment in physical activity programming and the use of recreational spaces following the pandemic. Specifically, as COVID-19 protocols lessen, steps need to be taken to safely return families to recreational places, including implementing facility and activity-specific health protocols, providing outdoor activity options, and offering a variety of activity types, times, and locations. Additional studies are needed to assess the transmission of COVID-19 in recreational facilities and identify protocols that improve children’s safety to facilitate children’s return to physical activity programming.

## Supplementary Information

**Additional file 1.**

## Data Availability

The datasets generated and/or analysed during the current study are not publicly available due to research ethics board requirements, but are available from the corresponding author on reasonable request.
