# Supplementary material for: Parents’ perceptions of their children’s physical activity during the COVID-19 pandemic
Source: BMC Public Health. 2022 Aug 1;22:1459. doi: 10.1186/s12889-022-13829-y (PMC9340749; doi:10.1186/s12889-022-13829-y)
Supplement: Supplementary file 1 — Additional file 1. [file 12889_2022_13829_MOESM1_ESM.docx]

**Additional File 1**: COVID-19 and Children’s Physical Activity Parent Interview Guide

**Welcome:**

Thank you for taking the time to participate in an interview discussing your child’s physical activity. My name is __[interviewer name]___ and I am ___[affiliation]___. Before we start, I wanted to check that you are still okay with our conversation being audio-recorded.

**Overview:**

 Today, we’d like to hear about your  family’s experiences during the COVID-19 pandemic to understand if your child’s engagement in physical activity has been affected and how we can support your child’s return to physical activity programming in local community centres, pools, areas, and small businesses. The discussions from this interview will be provide suggestions for program co-ordinators on ways programming can be altered in order to assist in children’s return to physical activity in public places.

As a reminder...

***Physical activity*** is any activity that increases your child’s heart rate and causes them to be out of breath. Physical activity can take place while playing sports, doing school activities, playing with friends, or walking to school.

**Guidelines:**

- There are 6 questions and should take around 15-20 minutes
- Our conversation today will be audio-recorded, and I will be taking notes throughout the discussion
- Your identity will remain confidential
- As we are asking about your personal experiences and thoughts, there are no right or wrong answers. If you are not comfortable with answering one of the questions, please let me know and we can move onto the next question.

At the end of the interview, we will review what we talked about and there will be time to add any ideas you may have

**Introduction:**

As a warm up: Can you tell me a little bit about your family?

1. Tell us about your child’s physical activity over the last month (e.g., Stage 3 COVID Return)

- How active is your child?
- What are they doing?
- Who are they with?
- Where are they doing it?

1. How has your child’s physical activity changed since March, when the pandemic started?

- Talk about July – Aug (“throughout the summer months”)
- Talk about April – June (maybe describe instead of use months to help trigger memories... “when COVID-19 safety regulations initially closed schools and businesses, around April to June, ”)
- How active is your child?
- What are they doing?
- Who are they with?
- Where are they doing it?

1. How have the public health measures/requirements influenced your child’s physical activity levels?

- Why has it increased / decreased?
- Why are the activities different?
- Why have they changes / stay the same?

1. How do you anticipate your child’s activity levels changing in the next 4-6 months?

- Will your child be more / less active than currently?
- Will their activities change than currently?
- Will they go more places than currently?

1. How can the (a) ACT-i-Pass / (b) City of London, YMCA, Boys & Girls Club and other local service providers, support your child’s physical activity and well-being this year?

- What type of programs would you like your child to have access to?
- What would make programs being offered more fun for you child?
- What could a service provider do to make you and your child more comfortable to use their facility and programs?

1. What have you and your child missed since COVID started?

- Are there specific places missed? Activities?

Quick review of what was discussed...

1. Are there any points or ideas that you would like to add before we end the interview?
